# Hyperperfusion Improvement: A Potential Therapeutic Marker in Neuromyelitis Optica Spectrum Disorder (NMOSD)

**DOI:** 10.3390/diagnostics15212723

**Published:** 2025-10-27

**Authors:** Koichi Kimura, Koji Hayashi, Mamiko Sato, Yuka Nakaya, Asuka Suzuki, Naoko Takaku, Hiromi Hayashi, Kouji Hayashi, Toyoaki Miura, Yasutaka Kobayashi

**Affiliations:** 1 Department of Rehabilitation Medicine, Fukui General Hospital, 55-16-1 Egami-cho, Fukui 910-8561, Japan; 2 Graduate School of Health Science, Fukui Health Science University, 55-13-1 Egami, Fukui 910-3190, Japan; 3 Department of Neurology, University of Fukui Hospital, 23-3 Matsuoka Shimoaizuki, Eiheiji-cho, Yoshida-gun, Fukui 910-1193, Japan

**Keywords:** neuromyelitis optica spectrum disorder, hyperperfusion, arterial spin labeling, prognosis, MRI

## Abstract

A 70-year-old Japanese woman with longstanding hearing loss and asthma developed floating sensations, left finger numbness, and postural instability one day after influenza vaccination, leading to hospital admission. Neurological examinations showed hearing loss, hyperreflexia, left-predominant ataxia, bilateral mild bathyanesthesia, and inability to tandem gait. Cerebrospinal fluid (CSF) analysis showed no pleocytosis or malignant cells, but revealed positive oligoclonal bands and elevated myelin basic protein. Despite no contrast agent use due to asthma, brain magnetic resonance imaging (MRI) revealed pontine hyperintensities on diffusion-weighted imaging (DWI) and T2-fluid attenuated inversion recovery (T2-FLAIR) sequences, along with hyperperfusion on arterial spin labeling (ASL) imaging. Serum anti-aquaporin-4 antibodies (AQP4-Ab) were negative by ELISA. Given the temporal proximity to vaccination and elevated demyelination markers, brainstem-type acute disseminated encephalomyelitis (ADEM) was initially suspected. Symptoms nearly resolved after two cycles of methylprednisolone pulse therapy. Notably, hyperperfusion gradually improved on ASL imaging. Post-discharge, a cell-based assay confirmed the diagnosis of neuromyelitis optica spectrum disorder (NMOSD) by detecting positive anti-AQP4-Ab. She has been relapse-free for about a year without any immunosuppressants or biologics. Although contrast-enhanced MRI remains the gold standard modality for lesion evaluation due to its high sensitivity, hyperperfusion on ASL may provide a useful alternative in patients for whom contrast agents are contraindicated, such as those with asthma or impaired renal function.

**Figure 1:**
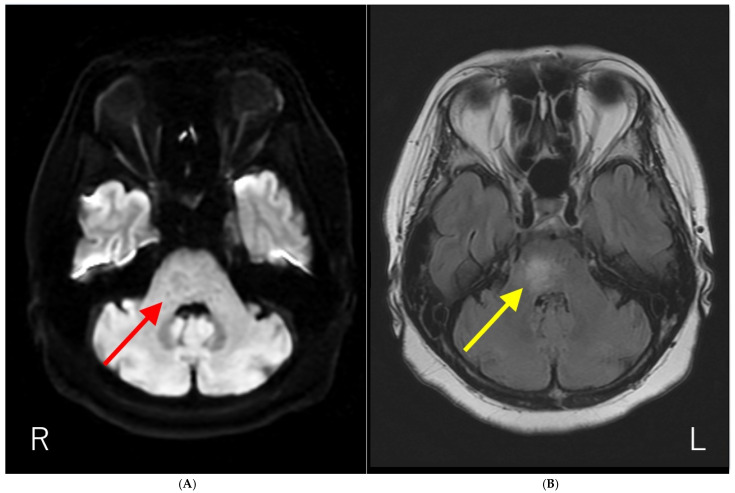
Brain magnetic resonance imaging (MRI) results. (**A**) Diffusion-weighted brain MRI showing ring-like hyperintensity in the pons (red arrow). (**B**) T2-fluid attenuated inversion recovery imaging (T2-FLAIR) showing hyperintensity with fuzzy borders in the pons (yellow arrow). The patient had a history of influenza vaccination immediately before the development of neurological symptoms. In addition, the anti-AQP4-Ab test by ELISA methods was negative. Initially, we suspected acute disseminated encephalomyelitis (ADEM) based on the temporal proximity of vaccination and neurological symptoms. Subsequently, anti-AQP4-Ab were detected using a cell-based assay, and the patient was diagnosed with neuromyelitis optica spectrum disorder (NMOSD). NMOSD is a group of autoantibody-mediated chronic inflammatory central nervous system (CNS) diseases that are distinct from multiple sclerosis (MS) [1,2]. Historically, it was initially described by Devic in 1894 as “neuro-myélite optique aiguë,” which translates to “neuromyelitis optica acuta,” after he described a case of bilateral blindness and paraplegia [1,2]. For much of the 20th century, the diagnosis was typically made in patients with a monophasic syndrome of severe, nearly simultaneous optic neuritis and myelitis [3]. A major breakthrough occurred in 2004 with the discovery of serum autoantibodies (initially called NMO-IgGs) specific for NMO, which were subsequently shown to target the astrocytic water channel aquaporin-4 (AQP4) in 2005 [2,3]. The detection of these anti-AQP4-Ab is now a central component of diagnostics in NMOSD [1]. This discovery expanded the understanding of the disease, leading to the broader term “NMOSD” to encompass a spectrum of clinical symptoms and MRI findings associated with anti-AQP4-Ab seropositivity [4,5]. Brain lesions are common but often asymptomatic, with typical locations including periependymal surfaces of the third and fourth ventricles, area postrema, corpus callosum, hypothalamus, and thalamus [1,2,3]. As far as we know, the report of the solitary pons lesion in NMOSD is limited [6].

**Figure 2:**
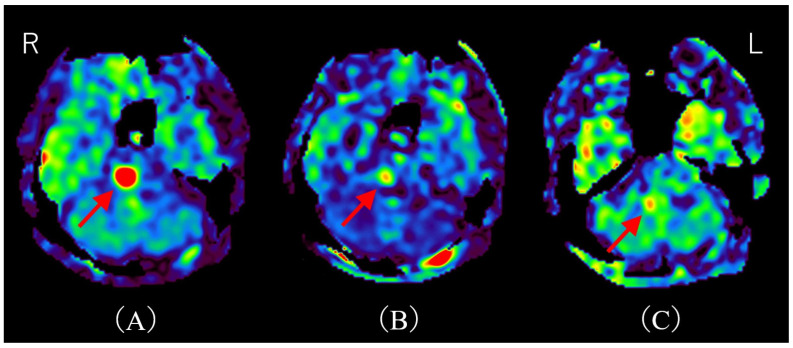
Time course of MRI on arterial spin labeling (ASL). (**A**) ASL upon admission shows hyperperfusion, consistent with the T2-FLAIR hyperintensity area (red arrow). (**B**) ASL on day 11 post-admission (after two courses of methylprednisolone pulse therapy) demonstrates marked improvement in hyperperfusion (red arrow). (**C**) ASL on day 32 post-admission reveals almost complete resolution of hyperperfusion (red arrow). R; right, L; left. There have been several reports of hyperperfusion in demyelinating cerebral lesions. Shibahara et al. reported hyperperfusion in myelin oligodendrocyte glycoprotein–associated disorders (MOGAD) [5]. Interestingly, they describe hyperperfusion correlating with disease activity, and hyperperfusion improves after steroid therapy. Yeo et al. reported regional hypoperfusion and focal central hyperperfusion lesions in Balo’s concentric sclerosis [6]. They note that cerebral blood flow (CBF) may be increased during acute demyelination due to disruption of the blood–brain barrier (BBB), and decreased in chronic demyelination due to axonal loss and regional hypometabolism. Khoury et al. reported hyperperfusion in progressive multifocal leukoencephalopathy (PML) lesions [7]. They found that a higher fraction of hyperperfused lesions is significantly associated with PML progression. Notably, this mechanism appears independent of BBB disruption, since hyperperfusion in PML lesions is generally not associated with contrast enhancement [7]. Instead, they speculated that nitric oxide, a potent vasodilator, mediates this hyperperfusion. Thus, although the precise mechanism of hyperperfusion in acute demyelinating lesions remains unclear, we hypothesize that BBB disruption caused by anti-AQP4-Ab may underlie the hyperperfusion observed in our case. Indeed, an in vitro model demonstrated that NMOSD patient-derived AQP4-Ab induces IL-6 production in astrocytes; this IL-6 then impairs endothelial barrier function and increases inflammatory chemokine expression, ultimately disrupting BBB tight junctions [8]. Additionally, consistent with previously reported cases of MOGAD, hyperperfusion served as a treatment marker in our patient. This observation is particularly significant, suggesting the potential utility of ASL as a non-invasive biomarker for assessing treatment response in NMOSD. While our study hypothesizes that hyperperfusion detected by ASL is primarily due to disruption of the BBB induced by anti-AQP4 antibodies, it is important to acknowledge alternative and potentially complementary mechanisms contributing to increased perfusion in demyelinating lesions. Reactive inflammation involving activated microglia and astrocytes is known to increase local metabolic demand, thereby elevating CBF independently of BBB disruption [9,10]. For example, glial activation can release vasodilatory mediators such as nitric oxide and cytokines, which cause vasodilation and enhanced perfusion [7,11,12,13]. This inflammatory hyperemia reflects an energy-demanding reparative or immune response rather than simple barrier leakage. Moreover, metabolic hyperactivity in inflamed tissue may heighten oxygen and glucose consumption, driving regional hyperperfusion to meet these energetic needs [14,15]. Such mechanisms have been described in multiple sclerosis and other demyelinating disorders, where focal perfusion increases correlate with active inflammation and gliosis [9,14]. Therefore, the observed ASL hyperperfusion in our NMOSD patient might represent a combination of BBB impairment allowing plasma leakage and inflammatory-metabolic factors elevating CBF, rather than a singular process. Notably, the patient underwent methylprednisolone pulse therapy, which resulted in the improvement of her symptoms. This clinical improvement correlated with a notable reduction in hyperperfusion observed on follow-up ASL imaging. Although contrast-enhanced MRI remains the gold standard modality for lesion evaluation due to its high sensitivity [16], hyperperfusion on ASL may provide a useful alternative in patients for whom contrast agents are contraindicated, such as those with asthma or impaired renal function. This report offers two distinct and valuable contributions to the existing literature. The first is the documentation of a rare presentation involving hyperperfusion. This article highlights a relatively uncommon manifestation of NMOSD, characterized by a solitary pontine lesion exhibiting hyperperfusion detected via ASL imaging. This specific finding adds to the body of knowledge regarding the atypical radiological presentations of NMOSD and provides valuable clinical insights into its diagnosis. The second contribution is the suggestion of ASL as a non-invasive biomarker for treatment response. Notably, this case indicates the potential utility of ASL imaging in assessing treatment efficacy in NMOSD, as changes in perfusion may serve as an objective measure of therapeutic response. Moreover, ASL offers an advantage as a non-invasive monitoring tool, especially for patients in whom contrast-enhanced MRI is contraindicated. Future work should validate ASL perfusion as a reliable, non-invasive biomarker across larger NMOSD cohorts and in longitudinal studies to establish its role in routine clinical management and monitoring.

## Data Availability

The data presented in this study are available on request from the corresponding author. Due to patient privacy and ethical considerations, the data are not publicly accessible.

## Abbreviations

The following abbreviations are used in this manuscript:

MRI	Magnetic resonance imaging
BBB	Blood–brain barrier
NMOSD	neuromyelitis optica spectrum disorder
ASL	Arterial spin labeling
MOGAD	Myelin oligodendrocyte glycoprotein–associated disorders
CBF	Cerebral blood flow
MS	Multiple sclerosis
PML	Progressive multifocal leukoencephalopathy
AQP4-Ab	Aquaporin-4 antibodies
